# A Robust Deep-Learning-Based Detector for Real-Time Tomato Plant Diseases and Pests Recognition

**DOI:** 10.3390/s17092022

**Published:** 2017-09-04

**Authors:** Alvaro Fuentes, Sook Yoon, Sang Cheol Kim, Dong Sun Park

**Affiliations:** 1 Department of Electronics Engineering, Chonbuk National University, Jeonbuk 54896, Korea; afuentes@jbnu.ac.kr; 2 Research Institute of Realistic Media and Technology, Mokpo National University, Jeonnam 534-729, Korea; syoon@mokpo.ac.kr; 3 Department of Computer Engineering, Mokpo National University, Jeonnam 534-729, Korea; 4 National Institute of Agricultural Sciences, Suwon 441-707, Korea; sckim@rda.go.kr; 5 IT Convergence Research Center, Chonbuk National University, Jeonbuk 54896, Korea

**Keywords:** plant disease, pest, deep convolutional neural networks, real-time processing, detection

## Abstract

Plant Diseases and Pests are a major challenge in the agriculture sector. An accurate and a faster detection of diseases and pests in plants could help to develop an early treatment technique while substantially reducing economic losses. Recent developments in Deep Neural Networks have allowed researchers to drastically improve the accuracy of object detection and recognition systems. In this paper, we present a deep-learning-based approach to detect diseases and pests in tomato plants using images captured in-place by camera devices with various resolutions. Our goal is to find the more suitable deep-learning architecture for our task. Therefore, we consider three main families of detectors: Faster Region-based Convolutional Neural Network (Faster R-CNN), Region-based Fully Convolutional Network (R-FCN), and Single Shot Multibox Detector (SSD), which for the purpose of this work are called “deep learning meta-architectures”. We combine each of these meta-architectures with “deep feature extractors” such as VGG net and Residual Network (ResNet). We demonstrate the performance of deep meta-architectures and feature extractors, and additionally propose a method for local and global class annotation and data augmentation to increase the accuracy and reduce the number of false positives during training. We train and test our systems end-to-end on our large Tomato Diseases and Pests Dataset, which contains challenging images with diseases and pests, including several inter- and extra-class variations, such as infection status and location in the plant. Experimental results show that our proposed system can effectively recognize nine different types of diseases and pests, with the ability to deal with complex scenarios from a plant’s surrounding area.

## 1. Introduction

Crops are affected by a wide variety of diseases and pests, especially in tropical, subtropical, and temperate regions of the world [1]. Plant diseases involve complex interactions between the host plant, the virus, and its vector [2]. The context of this problem is sometimes related to the effects of the climate change in the atmosphere and how it alters an ecosystem. Climate change basically affects regional climate variables, such as humidity, temperature, and precipitation, that consequently serve as a vector in which pathogens, virus, and plagues can destroy a crop, and thus cause direct impacts on the population, such as economic, health, and livelihood impacts [3].

Diseases in plants have been largely studied in the scientific area, mainly focusing on the biological characteristics of diseases [4]. For instance, studies on potato [5] and tomato [6,7] show how susceptible a plant is to be affected by diseases. The problem of plant diseases is a worldwide issue also related to food security [8]. Regardless of frontiers, media, or technology, the effects of diseases in plants cause significant losses to farmers [9]. An earlier identification of disease is nowadays a challenging approach and needs to be treated with special attention [10].

In our approach, we focus on the identification and recognition of diseases and pests that affect tomato plants. Tomato is economically the most important vegetable crop worldwide, and its production has been substantially increased through the years [11]. The worldwide cultivation of tomato exposes the crop to a wide range of new pathogens. Many pathogens have found this crop to be highly susceptible and essentially defenseless [6]. Moreover, viruses infecting tomato have been described, while new viral diseases keep emerging [12].

Several techniques have been recently applied to apparently identify plant diseases [13]. These include using direct methods closely related to the chemical analysis of the infected area of the plant [14,15,16], and indirect methods employing physical techniques, such as imaging and spectroscopy [17,18], to determine plant properties and stress-based disease detection. However, the advantages of our approach compared to most of the traditionally used techniques are based on the following facts:

- Our system uses images of plant diseases and pests taken in-place, thus we avoid the process of collecting samples and analyzing them in the laboratory.
- It considers the possibility that a plant can be simultaneously affected by more than one disease or pest in the same sample.
- Our approach uses input images captured by different camera devices with various resolutions, such as cell phone and other digital cameras.
- It can efficiently deal with different illumination conditions, the size of objects, and background variations, etc., contained in the surrounding area of the plant.
- It provides a practical real-time application that can be used in the field without employing any expensive and complex technology.

**Figure 1:**
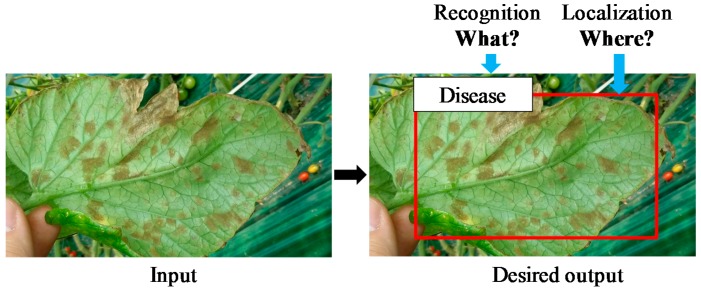
Recognition and localization of plant diseases and pests: problem formulation. Our system aims to detect both class (what) and location (where) of the affected areas in the image.

Plant diseases visibly show a variety of shapes, forms, colors, etc. [10]. Understanding this interaction is essential to design more robust control strategies to reduce crop damage [2]. Moreover, the challenging part of our approach is not only in disease identification but also in estimating how precise it is and the infection status that it presents. At this point, it is necessary to clarify the differences between the notions of image classification and object detection. Classification estimates if an image contains any instances of an object class (what), unlike a detection approach, which deals with the class and location instances of any particular object in the image (what and where). As shown in Figure 1, our system is able to estimate the class based on the probability of a disease and its location in the image shown as a bounding box containing the infected area of the plant.

Recent advances in hardware technology have allowed the evolution of Deep Convolutional Neural Networks and their large number of applications, including complex tasks such as object recognition and image classification. Since the success of AlexNet [19] in the ImageNet Large Scale Visual Recognition Challenge [20] 2012 (ILSVRC), deeper and deeper networks [21,22,23,24,25,26] have been proposed and achieved state-of-the-art performance on ImageNet and other benchmark datasets [27]. Thus, these results evidence the need to study the depth and width, as deeper and wider networks generate better results [28].

In this paper, we address disease and pest identification by introducing the application of deep meta-architectures [29] and feature extractors. Instead of using traditionally employed methods, we basically develop a system that successfully recognizes different diseases and pests in images collected in real scenarios. Furthermore, our system is able to deal with complex tasks, such as infection status (e.g., earlier, last), location in the plant (e.g., leaves, steam), sides of leaves (e.g., front, back), and different background conditions, among others.

Following previous approaches [30,31,32], we aim to use meta-architectures based on deep detectors to identify Regions of Interest (ROI) in the image, which correspond to infected areas of the plant. Each ROI is then classified as containing or not containing a disease or pest compared to the ground-truth annotated data. Using deep feature extractors, our meta-architecture can efficiently learn complex variations among diseases and pests found in different parts of the plant and deal with different sizes of candidates in the image.

The contributions of this paper are as follows: we propose a robust deep-learning-based detector for real-time tomato diseases and pests recognition. The system introduces a practical and applicable solution for detecting the class and location of diseases in tomato plants, which in fact represents a main comparable difference with traditional methods for plant diseases classification. Our detector uses images captured in-place by various camera devices that are processed by a real-time hardware and software system using graphical processing units (GPUs), rather than using the process of collecting physical samples (leaves, plants) and analyzing them in the laboratory. Furthermore, it can efficiently deal with different task complexities, such as illumination conditions, the size of objects, and background variations contained in the surrounding area of the plant. A detailed review of traditional methods for anomaly detection in plants and deep-learning techniques is presented in Section 2. Our proposed deep-learning-based system and the process for detecting diseases and pests is detailed in Section 3. In Section 4, we show the experimental results to demonstrate how our detector is able to successfully recognize nine different diseases and pests and their location in the images while providing robust real-time results. Moreover, we found out that using a technique-based data annotation and augmentation method results in better performance. In the last section, we study some of the detection failures and conclude that, although the system shows outstanding performance when dealing with all complex scenarios, there is still room for prediction improvements as our dataset becomes larger and includes more classes.

## 2. Related Works

### 2.1. Anomaly Detection in Plants

Plant diseases identification is a critical topic that has been studied through the years, and is motivated by the need to produce healthy food. However, some desirable elements to take into account should be cost-effectiveness, user-friendliness, sensitiveness, and accuracy [33]. In the last decade, several works have proposed some nondestructive techniques to overcome those facts. In [34], hyperspectral proximal sensing techniques were used to evaluate plant stress to environmental conditions. Optical technologies are practical tools considered for monitoring plant health; for example, in [35], thermal and fluorescence imaging methods were introduced for estimating plant stress produced mainly by increased gases, radiation, water status, and insect attack, among others. Another important area includes the study of plant defense in response to the presence of pathogens. For that effect, in [36], chemical elements were applied to leaves in order to estimate their defense capabilities against pathogens. To study plant robustness against nutritional facts, in [37], potato plants were cultivated in the presence of several nutritional elements to evaluate their effects in the crop.

As mentioned earlier, the area of plant anomaly detection has been dealt with by different media. Although previous methods show outstanding performance in the evaluated scenarios, they do not provide yet a highly accurate solution for estimating diseases and pests in a real-time manner. Instead, their experiments are mainly conducted in a laboratory or using expensive techniques. Therefore, our approach is focused on a cost-effective technique that uses images collected in situ as our source of information, including variations of the scenario in place. Before Deep Learning became popular in the Computer Vision field, several handcrafted feature-based methods had been widely applied specifically for image recognition. A handcrafted method is called so because of all the human knowledge implied in the development of the algorithm itself and the complex parameters that are included in the process. Some disadvantages of these methods are also the high computational cost and time consumption due to the complex preprocessing, feature extracting, and classifying. Some of the best-known handcrafted feature methods are the Histogram of Oriented Gradients (HOG) [38] and Scale-Invariant Feature Transform (SIFT) [39], which are usually combined with classifiers such as Adaptive Boosting (AdaBoost) [40] or Support Vector Machines (SVM) [41].

The facilities of Deep Learning have allowed researchers to design systems that can be trained and tested end-to-end (all included in the same process), unlike when using handcrafted-based methods that use separate processes. Due to the outstanding performance of Convolutional Neural Networks (CNNs) as a feature extractor in image recognition tasks, the idea has been also extended to different areas, such as in agriculture, automation, and robotics. Some of the applications for agriculture utilize Computer Vision and CNNs to solve complex tasks, such as plant recognition. For instance, in [42], it is shown how a CNN-based method outperforms local feature descriptors and bag of visual words techniques when recognizing 10 types of plants. In [43], the authors found that using a fusion of deep representations and handcrafted features leads to a higher accuracy of leaf plant classification. They applied a CNN for leaf segmentation, extracted handcrafted features with image processing techniques, trained an SVM with feature vectors, and used an SVM with a CNN to identify species among 57 varieties of trees.

Subsequently, due to the recent advance in Machine Learning, the principle of CNN has been applied to plant diseases recognition in different crops, such as [44] using a CNN-based LeNet and image processing to recognize two leaf diseases out of healthy ones. In [45], an image processing and statistical inference approach was introduced to identify three types of leaf diseases in wheat. In [46], the authors developed a method to discriminate good and bad condition images which contain seven types of diseases out of healthy ones in cucumber leaves. For that effect, they used an image-processing technique and a four-layer CNN, which showed an average of 82.3% accuracy under a 4-fold cross-validation strategy. Another approach for cucumber leaf diseases, [47], used a three-layer CNN to train images containing two diseases out of healthy ones. To support the application of machine learning, [48] proposed to use a method called Color and Oriented FAST and Rotated BRIEF (ORB) to extract features and tree classifiers (Linear Support Vector Classifier (SVC), K-Nearest Neighbor, Extremely Randomized Trees) to recognize four types of diseases in cassava. As a result, they present a smartphone-based system that uses the classification model that has learned to do real-time prediction of the state of health of a farmer’s garden.

Other works that use deep convolutional neural networks for diseases recognition have been also proposed, showing good performance on different crops. For instance, [49] developed a CNN-based system to identify 13 types of diseases out of healthy ones in five crops using images downloaded from the internet. The performance of that approach shows a top-1 success of 96.3% and top-5 success of 99.99%. In [50], the authors evaluate two CNN approaches based on AlexNet [19] and GoogleNet [23], to distinguish 26 diseases included in 14 crops using the Plant Village Dataset [51]. Another work in the same dataset shows a test accuracy of 90.4% using a VGG-16 model trained with transfer learning [52]. However, the Plant Village Dataset contains only images of leaves that are previously cropped in the field and captured by a camera in the laboratory. This is unlike the images in our Tomato Diseases and Pest Dataset, which are directly taken in-place by different cameras with various resolutions, including not only leaves infected by specific pathogens at different infection stages but also other infected parts of the plant, such as fruits and stems. Furthermore, the challenging part of our dataset is to deal with background variations mainly caused by the surrounding areas or the place itself (greenhouse).

Although the works mentioned above show outstanding performance on leaf diseases recognition, the challenges, such as pattern variation, infection status, different diseases or pests and their location in the image, and surrounding objects, among others, are still difficult to overcome. Therefore, we consider a technique that not only recognizes the disease in the image but also identifies its location for the posterior development of a real-time system.

### 2.2. Deep Meta-Architectures for Object Detection

Convolutional Neural Networks are considered nowadays as the leading method for object detection. As hardware technology has been improved through the years, deeper networks with better performance have been also proposed. Among them, we mention some state-of-the-art methods for object recognition and classification. In our paper, we focus principally on three recent architectures: Faster Region-Based Convolutional Neural Network (Faster R-CNN) [30], Single Shot Multibox Detector (SSD) [31], and Region-based Fully Convolutional Networks (R-FCN) [32]. As proposed in [29], while these meta-architectures were initially proposed with a particular feature extractor (VGG, Residual Networks ResNet, etc.), we now apply different feature extractors for the architectures. Thus, each architecture should be able to be merged with any feature extractor depending on the application or need.

#### 2.2.1. Faster Region-based Convolutional Neural Network (Faster R-CNN)

In Faster R-CNN, the detection process is carried out in two stages. In the first stage, a Region Proposal Network (RPN) takes an image as input and processes it by a feature extractor [30]. Features at an intermediate level are used to predict object proposals, each with a score. For training the RPNs, the system considers anchors containing an object or not, based on the Intersection-over-Union (IoU) between the object proposals and the ground-truth. In the second stage, the box proposals previously generated are used to crop features from the same feature map. Those cropped features are consequently fed into the remaining layers of the feature extractor in order to predict the class probability and bounding box for each region proposal. The entire process happens on a single unified network, which allows the system to share full-image convolutional features with the detection network, thus enabling nearly cost-free region proposals.

Since the Faster R-CNN was proposed, it has influenced several applications due to its outstanding performance on complex object recognition and classification.

#### 2.2.2. Single Shot Detector (SSD)

The SSD meta-architecture [31] handles the problem of object recognition by using a feed-forward convolutional network that produces a fixed-size collection of bounding boxes and scores for the presence of an object class in each box. This network is able to deal with objects of various sizes by combining predictions from multiple feature maps with different resolutions. Furthermore, SSD encapsulates the process into a single network, avoiding proposal generation and thus saving computational time.

#### 2.2.3. Region-based Fully Convolutional Network (R-FCN)

The R-FCN framework [32] proposes to use position-sensitive maps to address the problem of translation invariance. This method is similar to Faster R-CNN, but instead of cropping features from the same layer where region proposals are predicted, features (regions with a higher probability of containing an object or being part of it) are cropped from the last layer of features prior to prediction [29]. By the application of that technique, this method minimizes the amount of memory utilized in region computation. In the original paper [32], they show that using a ResNet-101 as feature extractor can generate competitive performance compared to Faster R-CNN.

### 2.3. Feature Extractors

In each meta-architecture, the main part of the system is the “feature extractor” or deep architecture. As mentioned in the previous section, year by year different deep architectures have been proposed and their application drastically depends on the complexity of problem itself. There are some conditions that should be taken into consideration when choosing a deep architecture, such as the type or number of layers, as a higher number of parameters increases the complexity of the system and directly influences the memory computation, speed, and results of the system.

Although each network has been designed with specific characteristics, all share the same goal, which is to increase accuracy while reducing computational complexity. In Table 1, some of the feature extractors used in this work are mentioned, including their number of parameters and performance achieved in the Image Net Challenge. We select some of the recent deep architectures because of their outstanding performance and applicability to our system.

**Figure 2:**
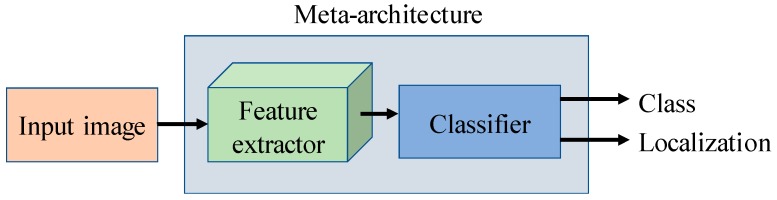
Flow chart of the deep meta-architecture approach used in this work. Our system proposes to treat a deep meta-architecture as an open system on which different feature extractors can be adapted to perform on our task. The system is trained and tested end-to-end using images captured in-place. The outputs are the class and localization of the infected area in the image.

As shown in Figure 2, our system proposes to treat the deep meta-architecture as an open system on which different feature extractors can be adapted to perform on our task. The input image captured by a camera device with different resolutions and scales is fed into our system, which after processing by our deep network (feature extractor and classifier) results in the class and localization of the infected area of the plant in the image. Thus, we can provide a nondestructive local solution only where the damage is presented, and therefore avoid the disease’s expansion to the whole crop and reduce the excessive use of chemical solutions to treat them.

## 3. Deep Meta-Architectures-Based Plant Diseases and Pest Recognition

### 3.1. System Background

Tomato plants are susceptible to several disorders and attacks caused by diseases and pests. There are several reasons that can be attributable to the effects on the crops: (1) abiotic disorders due to the environmental conditions, such as temperature, humidity, nutritional excess (fertilizer), light, and species of plant; (2) some pest that spread the disease from plant to plant, such as whiteflies, leaf miners, worms, bugs, etc; and (3) the most common diseases that include bacterial, virus, and fungal diseases. Those diseases and pests along with the plant may present different physical characteristics, such as a variety of shapes, colors, forms, etc. Therefore, due to similar patterns, those variations are difficult to be distinguished, which furthermore makes their recognition a challenge, and an earlier detection and treatment can avoid several losses in the whole crop.

Based on the facts above mentioned, we consider the following characteristics for our analysis:

- Infection status: A plant shows different patterns along with their infection status according to the life cycle of the diseases.
- Location of the symptom: It considers that diseases not only affect leaves, but also other parts of the plant such as stem or fruits.
- Patterns of the leaf: Symptoms of the diseases show visible variations either on the front side or the back side of the leaves.
- Type of fungus: Identifying the type of fungus can be an easy way to visibly differentiate between some diseases.
- Color and shape: Depending on the disease, the plant may show different colors or shapes at different infection stages.

**Figure 3:**
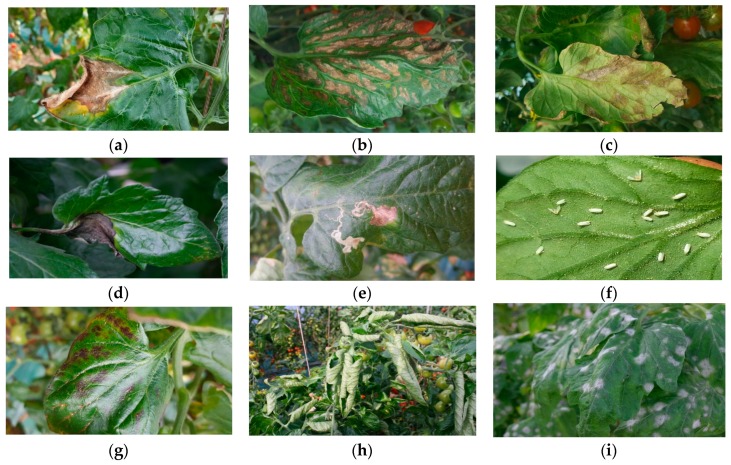
A representation of diseases and pests that affect tomato plants. (**a**) Gray mold, (**b**) Canker, (**c**) Leaf mold, (**d**) Plague, (**e**) Leaf miner, (**f**) Whitefly, (**g**) Low temperature, (**h**) Nutritional excess or deficiency, (**i**) Powdery mildew. The images are collected under different variations and environmental conditions. The patterns help to distinguish some proper characteristics of each disease and pest.

In Figure 3, we show a representation of the diseases and pests under different conditions and variations identified in our work. A detailed study of each disease’s and pest’s symptoms is described in [10].

### 3.2. System Overview

**Figure 4:**
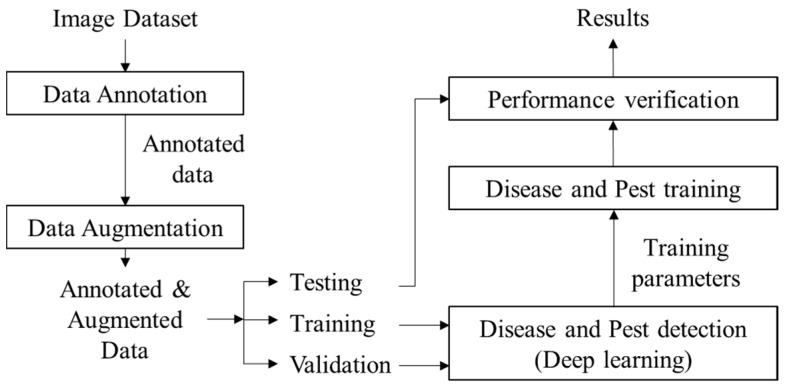
System overview of the proposed deep-learning-based approach for plant diseases and pest recognition. Our deep meta-architecture approach consists of several steps that use input images as a source of information, and provide detection results in terms of class and location of the infected area of the plant in the image.

Our work aims to identify nine classes of diseases and pests that affect tomato plants using Deep Learning as the main body of the system. A general overview of the system is presented in Figure 4. Following we describe in detail each component of the proposed approach.

### 3.3. Data Collection

Our dataset contains images with several diseases and pests in tomato plants. Using simple camera devices, the images were collected under several conditions depending on the time (e.g., illumination), season (e.g., temperature, humidity), and place where they were taken (e.g., greenhouse). For that purpose, we have visited several tomato farms located in Korea and fed our dataset with various types of data, including:

- Images with various resolutions.
- Samples at early, medium, and last infection status.
- Images containing different infected areas in the plant (e.g., stem, leaves, fruits, etc.).
- Different plant sizes.
- Objects surrounding the plant in the greenhouse, etc.

These conditions help to estimate the infection process and determine how a plant is affected by the disease or pest (origin or possible developing cause).

### 3.4. Data Annotation

Starting with the dataset of images, we manually annotate the areas of every image containing the disease or pest with a bounding box and class. Some diseases might look similar depending on the infection status that the present; therefore, the knowledge for identifying the type of disease or pest has been provided by experts in the area. That has helped us to visibly identify the categories in the images and infected areas of the plant.

This annotation process aims to label the class and location of the infected areas in the image. The outputs of this step are the coordinates of the bounding boxes of different sizes with their corresponding class of disease and pest, which consequently will be evaluated as the Intersection-over-Union (IoU) with the predicted results of the network during testing. To make it more clear, an example of an annotated bounding box can be visualized in Figure 1. The red box shows the infected areas of the plant, and parts of the background.

Since our images are collected in the field, many areas corresponding to the background could be included in the image, making the problem more challenging. Therefore, when collecting the images, we find out that the best way to get more precise information is to capture the samples containing the ROIs as the main part of the image. As previously presented in Figure 1, the problem formulation of recognition and localization of the infected part of the plant makes our system different from others that are basically focused only on classification.

### 3.5. Data Augmentation

Although Deep Neural Network systems have shown outstanding performance compared to traditional machine learning or computer vision algorithms, the drawback of these systems is the overfitting problem. Overfitting is often referred to the hyper-parameters selection, system regularization, or a number of images used for training. Following [19], data augmentation is necessary to pursue when the number of images in the dataset is not enough. We use several techniques that basically increase the number of images of our dataset. These techniques consist of geometrical transformations (resizing, crop, rotation, horizontal flipping) and intensity transformations (contrast and brightness enhancement, color, noise).

### 3.6. Disease and Pest Detection

We now describe our main method for detecting diseases and pests. Our goal is to detect and recognize the class and location of disease and pest candidates in the image. To detect our target, we need to accurately localize the box containing the object, as well as identify the class to which it belongs.

As shown in Figure 2, our proposed solution aims to overcome such a complex problem by a simple and accurate form. We extend the idea of a meta-architecture-based object detection framework to adapt it with different feature extractors that detect diseases and pests and localize their position in the image. For that purpose, we have considered three meta-architectures due to their high performance in object detection. In the following, we explain in detail each meta-architecture and feature extractor.

#### 3.6.1. Faster R-CNN

We extend the application of Faster R-CNN [30] for object recognition and its Region Proposal Network (RPN) to estimate the class and location of object proposals that may contain a target candidate. The RPN is used to generate the object proposals, including their class and box coordinates. Then, for each object proposal, we extract the features with an RoI Pooling layer and perform object classification and bounding-box regression to obtain the estimated targets.

#### 3.6.2. SSD

We follow the methodology described in [31]. SSD generates anchors that select the top most convolutional feature maps and a higher resolution feature map at a lower resolution. Then, a sequence of the convolutional layer containing each detection per class is added with spatial resolution used for prediction. Thus, SSD is able to deal with objects of various sizes contained in the images. A Non-Maximum Suppression method is used to compare the estimated results with the ground-truth.

#### 3.6.3. R-FCN

We follow the implementation of R-FCN [32] as another meta-architecture to perform our approach. Similar to Faster R-CNN, R-FCN uses a Region Proposal Network to generate object proposals, but instead of cropping features using the RoI pooling layer it crops them from the last layer prior to prediction. We used batch normalization for each feature extractor, and train end-to-end using an ImageNet Pretrained Network.

We have selected the feature extractors based on their performance and number of parameters from Table 1. These are VGG-16, ResNet 50-152, and ResNeXt-50 for Faster R-CNN, ResNet-50 for SSD, and ResNet-50 for R-FCN. To perform the experiments, we have adapted the feature extractors to the conditions of each meta-architecture. For instance, in Faster R-CNN, each feature extractor includes the RPN and features are extracted from the “conv5” layer of VGG-16, the last layer of the “conv4” block in ResNet 50-152, as well as from the “conv4” block in ReNeXt-50. In SSD, in contrast to the original work, we use ResNet-50 as its basis feature extractor. In R-FCN, ResNet-50 is used as its feature extractor and the features are extracted from the “conv4” block.

Our training objective is to reduce the losses between the ground-truth and estimated results, as well as to reduce the presence of false positives in the final results, by Non-Maximum Suppression (NMS) of each meta-architecture, which selects only candidates only with an IoU > 0.5 compared to their initial annotated ground-truth. The loss functions and bounding-box encoding used in this work are presented in Table 2.

## 4. Experimental Results

### 4.1. Tomato Diseases and Pests Dataset

Our dataset consists of about 5000 images collected from farms located in different areas of the Korean Peninsula. The images were taken under different conditions and scenarios. They include diseases that can develop depending on the season and variables such as temperature and humidity. Since not all diseases can be found all year round, but rather in seasons, the number of images corresponding to each class is different. The categories and the number of annotated samples used in our system can be seen in Table 3. The number of annotated samples corresponds to the number of bounding boxes labeled in the images after data augmentation. Every image contains more than one annotated sample depending on the infection areas of the plant, and the background class is collected as a transversal category (hard negatives) that is annotated in most of the images. The background class has been called so because it contains areas of the image that correspond to healthy parts of the plant and from the background itself, such as the structure of the greenhouse.

### 4.2. Experimental Setup

**Figure 5:**
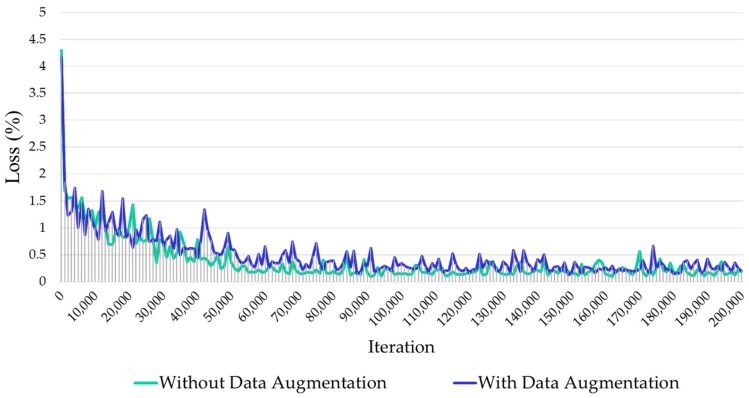
Training loss curve of our proposed approach. The comparison includes results with and without data augmentation. Our network efficiently learns the data while achieving a lower error rate at about one hundred thousand iterations.

We perform experiments on our Tomato Diseases and Pests dataset that includes nine annotated diseases and pest categories. As explained in the previous section, since the number of images in our dataset is still small and in order to avoid overfitting, we apply extensive data augmentation, including the techniques mentioned in Section 3.4. To perform the experiments, our dataset has been divided into 80% training set, 10% validation set, and 10% testing set. The training is proceeded on the training set, after that the evaluation is performed on the validation set, and when the experiments seem to achieve the expected results, the final evaluation is done on the testing set (unknown data). As in the Pascal Visual Object Classes (VOC) Challenge [53], the validation set is a technique used for minimizing overfitting and is a typical way to stop the network from learning. We use the training and validation sets to perform the training process and parameter selection, respectively, and the testing set for evaluating the results on unknown data. Our proposed system has been trained and tested end-to-end with an Intel Core I7 3.5 GHz Processor on two NVidia GeForce Titan X GPUs. Figure 5 illustrates the resultant loss curve for a number of two hundred thousand iterations, which demonstrates that our network efficiently learns the data while achieving a lower error rate at about one hundred thousand iterations.

### 4.3. Quantitative Results

Our proposed system implements meta-architectures and different feature extractors to deal with the detection and recognition of complex diseases and pests in the images. The performance of our system is evaluated first of all in terms of the Intersection-over-Union (IoU), and the Average Precision (AP) that is introduced in the Pascal VOC Challenge [53].

(1) $$IoU\left(A,B\right)=\left|\frac{A\cap B}{A\cup B}\right|$$

where *A* represents the ground-truth box collected in the annotation, and *B* represents the predicted result of the network. If the estimated *IoU* outperforms a threshold value, the predicted result is considered as a true positive, TP, or if not as a false positive, FP. TP is the number of true positives generated by the network, and FP corresponds to the number of false positives. Ideally, the number of FP should be small and determines how accurate is the network to deal with each case. The *IoU* is a widely used method for evaluating the accuracy of an object detector.

The Average Precision is the area under the Precision-Recall curve for the detection task. As in the Pascal VOC Challenge, the AP is computed by averaging the precision over a set of spaced recall levels [0, 0.1,…, 1], and the mAP is the AP computed over all classes in our task.

(2) $$AP=\frac{1}{11}\underset{r\in \left\{0,\;0.1,\ldots ,\;1\right\}}{\sum }p_{interp}\left(r\right)$$

(3) $$P_{interp}\left(r\right)=\underset{\tilde{r}:\tilde{r}\geq r}{\max}p\left(\tilde{r}\right)$$

where $p\left(\tilde{r}\right)$ is the measure precision at recall $\tilde{r}$. Next, we compute the mAP averaged for an *IoU* = 0.5 (due to the complexity of the scenarios). The detection results are shown in Table 4.

The comparative results show that, in our task, plain networks perform better than deeper networks, such as the case of Faster R-CNN with VGG-16 with a mean AP of 83%, compared to the same meta-architecture with ResNet-50 that achieves 75.37% or ResNeXt-50 with 71.1%. In contrast, SSD with ResNet-50 performs at 82.53% and R-FCN with ResNet-50 as feature extractor achieves a mean AP of 85.98%, which is slightly better than Faster R-CNN overall and is comparable in some classes.

Although the mean AP for the whole system shows a performance of more than 80% for the best cases, some diseases, such as leaf mold, gray mold, canker, and plague, show a variable performance. Both Faster R-CNN and R-FCN use the same method of Region Proposal Network (RPN) to extract features from the last layer of the CNN, but using different feature extractors as in our experiment, with VGG-16 and ResNet-50 for Faster R-CNN and R-FCN, respectively, shows comparable results and outstanding performance in the more challenging classes. An early estimation of diseases or pests in the plant could avoid several losses in the whole crop; therefore, we consider leaf mold, gray mold, canker, and pest as the most complex and main classes due to their high intra-class variation (e.g., infection status, location of the infection in the plant, side of leaf, type of fungus, etc.) and some inter-class similarities, especially in the last state of infection when the plant is already dead. Despite the complexity of the scenarios, Faster R-CNN with VGG-16 shows better recognition results especially on the classes above mentioned.

The number of samples is another fact that influences the generation of better results. That could be the case for leaf mold, since our dataset contains a number of samples of this class. The background class is a transversal category that is annotated in most of the images, including healthy parts of the plant as well as parts of the scenario. Furthermore, as we know, the implementation of deep learning systems requires a large number of data that can certainly influence the final performance. In Table 5, we show how the use of a data augmentation technique has allowed our system to improve the Average Precision for each case compared to a previously trained system without using data augmentation.

### 4.4. Qualitative Results

**Figure 6:**
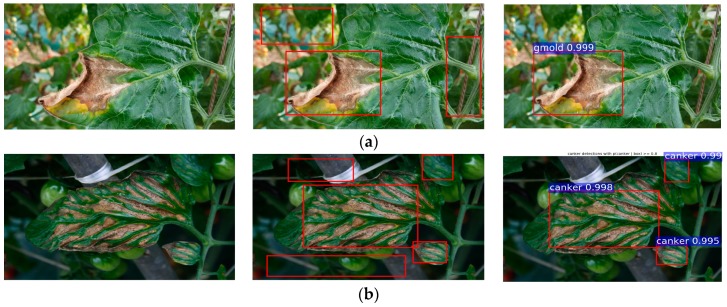

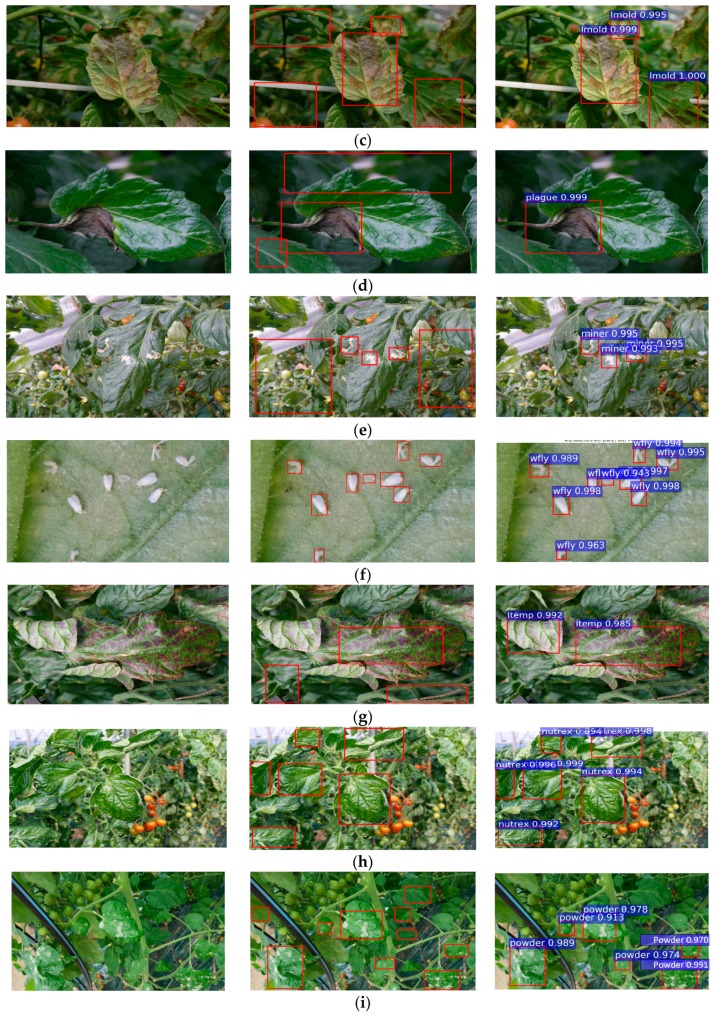
Detection results of diseases and pests that affect tomato plants with Faster R-CNN and a VGG-16 detector. From left to right: the input image, annotated image, and predicted results. (**a**) Gray mold; (**b**) Canker; (**c**) Leaf mold; (**d**) Plague; (**e**) Leaf miner; (**f**) Whitefly; (**g**) Low temperature; (**h**) Nutritional excess or deficiency; (**i**) Powdery mildew.

We evaluate the performance of bounding-box regression and the class score for each class in our Tomato Disease and Pest dataset. As shown in Figure 6, our system is able to effectively detect the class and location of diseases and pests. We compared the estimated results with the ground-truth using an *IoU* >0.5. Thus, the regions of interest can be estimated while avoiding false positives.

Each class is independent of each other, not only by its origin or cause but also visibly as they show different patterns and characteristics. We find the best results are generated when the main part of the image consists of the target candidate, in contrast with images that include large background regions.

Using meta-architectures and deep feature extractors, the system shows several advantages compared to previous traditional methods when dealing for instance with objects of various sizes (e.g., Gray mold vs. Whitefly), shapes (e.g., Leaf Mold vs. Canker), color (e.g., Plague vs. Leaf mold), etc. Moreover, the proposed approach introduces a fast and effective solution performing at about 160 ms per image.

### 4.5. Deep Network Visualization

Understanding a neural network can be interpreted as a deep analysis of how each neuron interacts in the learning process to generate the final results. For that effect, the most popular approach is to use Deconvolutional Neural Networks (DeConv). Using an input image, it aims to highlight which pixels in that image contribute to a neuron firing. This deconvolutional operation can be generated like a convolutional operation but in reverse, such as un-pooling feature maps and convolving un-pooled maps.

As mentioned earlier, diseases and pests in tomato plants can be produced by different causes, such as temperature, humidity, nutrients, lighting conditions, etc. At some point of their infection status, some diseases show similar characteristics or develop visible patterns in the plant that help to distinguish one from another. Therefore, by this experiment, we aim to find a feature map for each class which allows us to understand better their content and representation.

**Figure 7:**
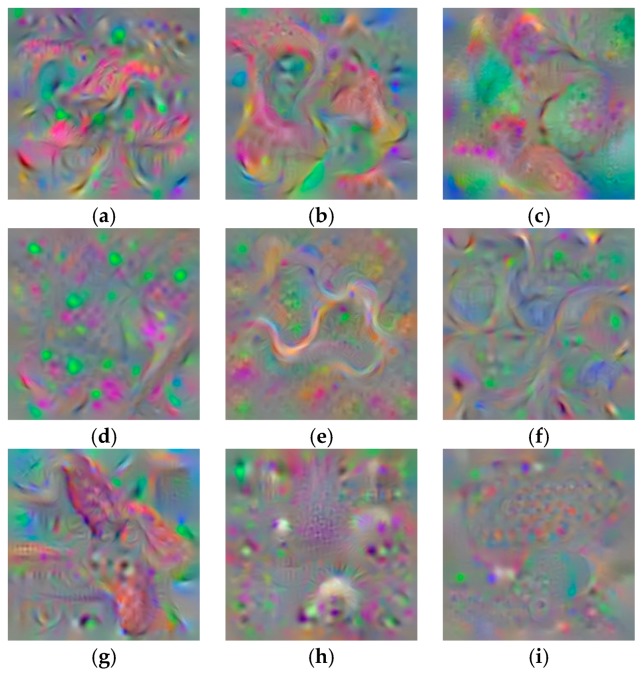
Deep feature maps visualization of diseases and pest (**a**) Canker; (**b**) Gray mold; (**c**) Leaf mold; (**d**) Low temperature; (**e**) Miner; (**f**) Nutritional excess; (**g**) Plague; (**h**) Powdery mildew; (**i**) Whitefly. Each feature map illustrates how our neural network system interprets a disease in the context after being classified by a SoftMax function.

After passing the images by a deconvolutional neural network, which is similar in structure to our main CNN but in a reverse procedure, the final representations are shown in Figure 7. Each feature map illustrates how our neural network system interprets a disease in the context after being classified by a SoftMax function.

### 4.6. Diseases Effects in the Plant

**Figure 8:**
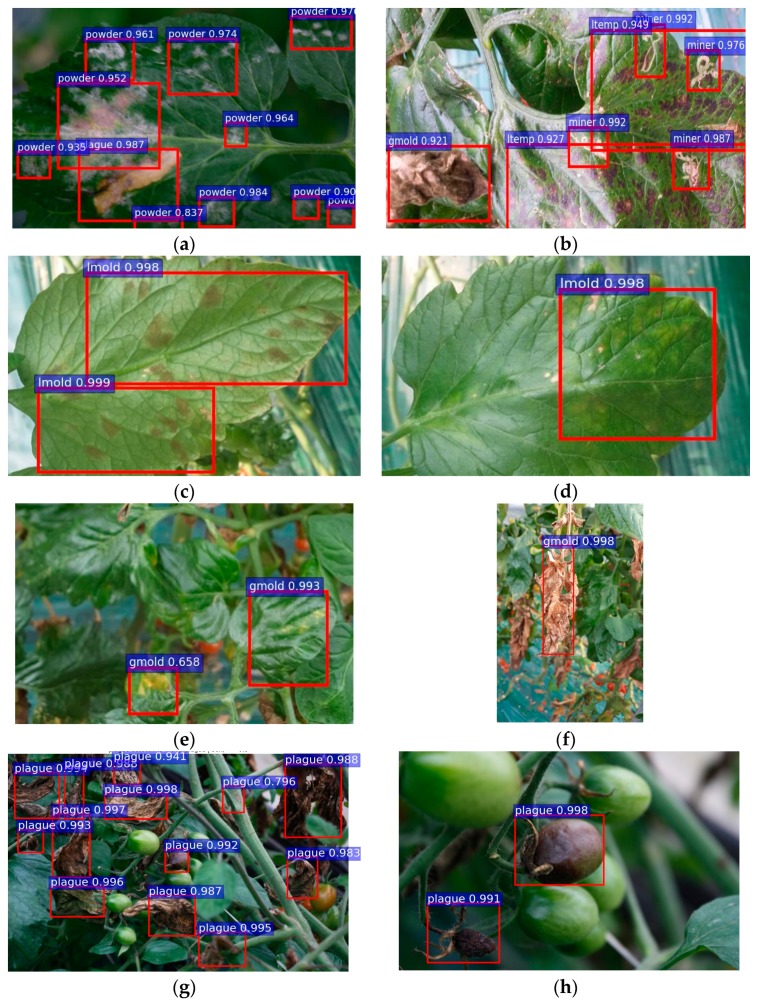
Detection results of inter- and intra-class variation of diseases and pests in the images. (**a**) Two classes affecting the same sample (powdery mildew and pest); (**b**) Three classes in the same sample (Gray mold, low temperature, and miners); (**c**) Leaf mold affecting the back side of the leaf; (**d**) Leaf mold affecting the front side of the leaf; (**e**) Gray mold in the early stage; (**f**) Gray mold in the last stage; (**g**) Plague can be also detected on other parts of the plant, such as fruits or stem; (**h**) Plague affecting the tomato production.

The infection symptoms of diseases and pests in the plants start by different ways. It could be started either by a disease originating in the plant itself or infection from other surrounding plants. Therefore, it is useful to identify all the possible causes affecting the plant in order to develop an early detection approach. As shown in Figure 8, diseases and pests can simultaneously affect a plant when it becomes vulnerable due to its condition. For example, Figure 8a shows how the effect of the white fungus, which is a characteristic of powdery mildew, appears to generate spot areas in the leaves where a plague can be developed easily. Furthermore, Figure 8b illustrates the detection results of low temperature, gray mold, and miners in the same plant. Figure 8c,d represent an example of intra-class variations, such as in leaf mold, where the sample leaf corresponds to the same class but with different patterns on its front side and back side. Figure 8e,f show the intra-class variations because of the infection status. Although both images belong to the same class, they visibly show different patterns at an early and the last stage, respectively. Figure 8g,h extend the idea of disease and pest identification to other parts of the plants, such as stem and fruits. Those are also special features that help to identify a pathogen affecting a plant. This experiment gives an idea of how our system is able to efficiently deal with inter- and intra-class variations and its importance as an early detection approach when the symptoms have just appeared.

### 4.7. Confusion Matrix

**Figure 9:**
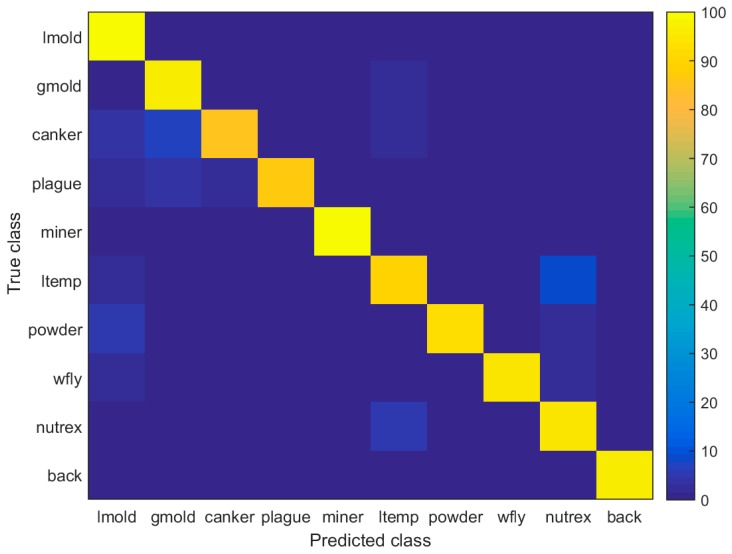
Confusion matrix of the Tomato Diseases and Pests detection results (including background, which is a transversal class containing healthy parts of the plants and surrounding areas, such as part of the greenhouse).

Due to the complexity of the patterns shown in each class, especially in terms of infection status and background, the system tends to be confused on several classes that results in lower performance. In Figure 9, we present a confusion matrix of the final detection results. Based on the results, we can visually evaluate the performance of the classifier and determine what classes and features are more highlighted by the neurons in the network. Furthermore, it helps us to analysis a further procedure in order to avoid those inter-class confusions. For instance, the canker class shows to be confused in more intensity with gray mold, but also with leaf mold and low temperature. Similarly, the low-temperature class shows confusion with the nutritional excess class.

### 4.8. Failures Analysis and Discussion

**Figure 10:**
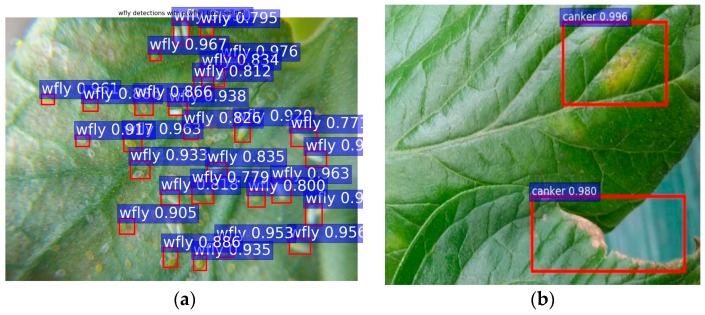
A representation of failure cases. (**a**) The intra-class variation makes the recognition harder and results in a low recognition rate. (**b**) Misdetected class due to confusion at earlier infection status (e.g., the real class is leaf mold, but the system recognizes it as canker).

Although our system shows an outstanding performance on the evaluated cases, it also presents difficulties in some cases that could be a possible topic for a further study. Due to the lacking number of samples, some classes with high pattern variation tend to be confused with others, resulting in false positives or lower average precision. As shown in Figure 10, for the white fly (e.g., eggs and whiteflies) and leaf mold classes, the presence of targets with different mature status makes their recognition hard when comparing the visible characteristics between them.

## 5. Conclusions

In this work, we have proposed a robust deep-learning-based detector for real-time tomato diseases and pests recognition. This system introduces a practical and applicable solution for detecting the class and location of diseases in tomato plants, which in fact represents a main comparable difference with other methods for plant diseases classification. Our detector applied images captured in-place by various camera devices and processed them by a real-time hardware and software system using GPUs, rather than using the process of collecting physical samples (leaves, plants) and analyzing them in the laboratory. Furthermore, our tomato plant diseases and pest dataset contains different task complexities, such as illumination conditions, the size of objects, background variations, etc., included in the surrounding area of the plant. Our goal was to find the more suitable deep-learning architecture for our task. Thus, the experimental results and comparisons between various deep-meta-architectures with feature extractors demonstrated how our deep-learning-based detector is able to successfully recognize nine different categories of diseases and pests, including complex intra- and inter-class variations. In addition, we found that using technique-based data annotation and augmentation results in better performance. We expect that our proposed system will make a significant contribution to the agriculture research area. Future works will be focused on improving the current results, and a promising application will be to extend the idea of diseases and pest recognition to other crops.

## Figures and Tables

**Table 1:** Properties of the deep feature extractors used in this work and their performance on the ImageNet Challenge.

Feature Extractor	Parameters (M)	Number of Layers	Top-5 Error
AlexNet [19]	61	8	15.3
ZFNet	-	8	14.8
VGG-16 [22]	138	16	7.40
GoogLeNet [23]	6.9	22	6.66
ResNet-50 [24]	25	50	3.57
ResNet-101 [24]	42.6	101	-
ResNetXt-101 [26]	42.6	101	3.03

**Table 2:** Details of Deep Learning Meta-architectures and Feature Extractors.

Meta-Architecture	Feature Extractor	Bounding Box	Loss Function
Faster R-CNN	VGG-16 ResNet-50 ResNet-101 ResNet-152 ResNeXt-50	$\left[\frac{x_{c}}{w_{a}},\frac{y_{c}}{h_{a}},\log w,\log h\right]$	Smooth$L_{1}$
SSD	ResNet-50	$\left[\frac{x_{c}}{w_{a}},\frac{y_{c}}{h_{a}},\log w,\log h\right]$	Smooth$L_{1}$
R-FCN	ResNet-50	$\left[\frac{x_{c}}{w_{a}},\frac{y_{c}}{h_{a}},\log w,\log h\right]$	Smooth$L_{1}$

Faster R-CNN: faster region-based convolutional neural network; SSD: single shot detector; R-FCN: region-based fully convolutional network.

**Table 3:** List of Categories included in Our Tomato Diseases and Pests Dataset and their Annotated Samples.

Class	Number of Images in the Dataset ^1^	Number of Annotated Samples (Bounding Boxes) ^2^	Percentage of Bounding Box Samples (%)
Leaf mold	1350	11,922	27.47
Gray mold	335	2768	6.37
Canker	309	2648	6.10
Plague	296	2570	5.92
Miner	339	2946	6.78
Low temperature	55	477	1.09
Powdery mildew	40	338	0.77
Whitefly	49	404	0.93
Nutritional excess	50	426	0.98
Background ^3^	2177	18,899	43.54
Total	5000	43,398	100

^1^ Number of images in the dataset; ^2^ Number of annotated samples after data augmentation; ^3^ Transversal category included in every image.

**Table 4:** Detection Results of Our Proposed System using Deep-Learning Meta-architectures and Feature Extractors.

Meta-Architectures							
	Faster R-CNN					R-FCN	SSD
Class/Feature Extractor	VGG-16	ResNet-50	ResNet-101	ResNet-152	ResNeXt-50	ResNet-50	ResNet-50
Leaf mold	**0.9060**	0.8827	0.803	0.8273	0.840	**0.8820**	0.8510
Gray mold	**0.7968**	0.6684	0.449	0.4499	0.620	**0.7960**	0.7620
Canker	**0.8569**	0.7580	0.660	0.7154	0.738	**0.8638**	0.8326
Plague	**0.8762**	0.7588	0.613	0.6809	0.742	**0.8732**	0.8409
Miner	0.8046	0.7884	0.756	0.7793	0.767	0.8812	0.7963
Low temperature	0.7824	0.6733	0.468	0.5221	0.623	0.7545	0.7892
Powdery mildew	0.6556	0.5982	0.413	0.4928	0.505	0.7950	0.8014
Whitefly	0.8301	0.8125	0.637	0.7001	0.720	0.9492	0.8402
Nutritional excess	0.8971	0.7637	0.547	0.8109	0.814	0.9290	0.8553
Background	0.9005	0.8331	0.624	0.7049	0.745	0.8644	0.8841
Total mean AP	**0.8306**	0.7537	0.590	0.6683	0.711	**0.8598**	0.8253

* The bold numbers correspond the more challenging classes and best results among other meta-architectures. AP: average precision.

**Table 5:** Influence of the Data Augmentation Technique in the Final Results ^1^.

Class	Without Data Augmentation	With Data Augmentation
Leaf mold	0.6070	0.9060
Gray mold	0.5338	0.7968
Canker	0.5741	0.8569
Plague	0.5870	0.8762
Miner	0.5390	0.8046
Low temperature	0.5242	0.7824
Powdery mildew	0.4392	0.6556
Whitefly	0.5591	0.8301
Nutritional excess	0.6010	0.8971
Background	0.6033	0.9005
Total mean AP	0.5564	0.8306

^1^ Experiments using the same meta-architecture and feature extractor (Faster R-CNN with VGG-16).

## References

[1] Mabvakure B., Martin D.P., Kraberger S., Cloete L., Van Bruschot S., Geering A.D.W., Thomas J.E., Bananej K., Lett J., Lefeuvre P. (2016). Ongoing geographical spread of Tomato yellow leaf curl virus. Virology.

[2] Canizares M.C., Rosas-Diaz T., Rodriguez-Negrete E., Hogenhout S.A., Bedford I.D., Bejarano E.R., Navas-Castillo J., Moriones E. (2015). Arabidopsis thaliana, an experimental host for tomato yellow leaf curl disease-associated begomoviruses by agroinoculation and whitefly transmission. Plant Pathol..

[3] The World Bank (2014). Reducing Climate-Sensitive Risks. http://documents.worldbank.org/curated/en/486511468167944431/Reducing-climate-sensitive-disease-risks.

[4] Nutter F.W., Esker P.D., Coelho R. (2006). Disease assessment concepts and the advancements made in improving the accuracy and precision of plant disease data. Eur. J. Plant Pathol..

[5] Munyaneza J.E., Crosslin J.M., Buchman J.L., Sengoda V.G. (2010). Susceptibility of Different Potato Plant Growth Stages of Purple Top Disease. Am. J. Potato Res..

[6] Gilbertson R.L., Batuman O. (2013). Emerging Viral and Other Diseases of Processing Tomatoes: Biology Diagnosis and Management. Acta Hortic..

[7] Diaz-Pendon J.A., Canizares M.C., Moriones E., Bejarano E.R., Czosnek H., Navas-Castillo J. (2010). Tomato yellow leaf curl viruses: Menage a trois between the virus complex, the plant and whitefly vector. Mol. Plant Pathol..

[8] Coakley S.M., Scherm H., Chakraborty S. (1999). Climate Change and Plant Disease Management. Annu. Rev. Phytopathol..

[9] Food and Agriculture Organization of the United Nations (2017). Plant Pests and Diseases. http://www.fao.org/emergencies/emergency-types/plant-pests-and-diseases/en/.

[10] Fuentes A., Yoon S., Youngki H., Lee Y., Park D.S. (2016). Characteristics of Tomato Plant Diseases—A study for tomato plant disease identification. Proc. Int. Symp. Inf. Technol. Converg..

[11] Food and Agriculture Organization of the United Nations (2015). Value of Agricultural Production-Tomatoes. Food and Agriculture data.

[12] Hanssen I., Lapidot M., Thomma B. (2010). Emerging Viral Diseases of Tomato Crops. Mol. Plant Microbe Interact..

[13] Sankaran S., Mishra A., Ehsani R. (2010). A review of advanced techniques for detecting plant diseases. Comput. Electron. Agric..

[14] Chaerani R., Voorrips R.E. (2006). Tomato early blight (Alternaria solani): The pathogens, genetics, and breeding for resistance. J. Gen. Plant Pathol..

[15] Alvarez A.M. (2004). Integrated approaches for detection of plant pathogenic bacteria and diagnosis of bacterial diseases. Annu. Rev. Phytopathol..

[16] Gutierrez-Aguirre I., Mehle N., Delic D., Gruden K., Mumford R., Ravnikar M. (2009). Real-time quantitative PCR based sensitive detection and genotype discrimination of Pepino mosaic virus. J. Virol. Methods.

[17] Martinelli F., Scalenghe R., Davino S., Panno S., Scuderi G., Ruisi P., Villa P., Stropiana D., Boschetti M., Goudart L. (2015). Advanced methods of plant disease detection. A review. Agron. Sust. Dev..

[18] Bock C.H., Poole G.H., Parker P.E., Gottwald T.R. (2007). Plant Disease Sensitivity Estimated Visually, by Digital Photography and Image Analysis, and by Hyperspectral Imaging. Crit. Rev. Plant Sci..

[19] Krizhenvshky A., Sutskever I., Hinton G. Imagenet classification with deep convolutional networks. Proceedings of the Conference Neural Information Processing Systems (NIPS).

[20] Russakovsky O., Deng J., Su H., Krause J., Satheesh S., Ma S., Huang Z., Karpathy A., Khosla A., Bernstein M. (2015). ImageNet Large Scale Visual Recognition Challenge. Int. J. Comput. Vis..

[21] Lin M., Chen Q., Yan S. (2013). Network in Network. arXiv.

[22] Simonyan K., Zisserman A. (2014). Very deep convolutional networks for large-scale image recognition. arXiv.

[23] Szegedy C., Liu W., Jia Y., Sermanet P., Reed S., Anguelov D., Erhan D., Vanhoucke V., Rabinovich A. Going deeper with convolutions. Proceedings of the 2015 IEEE Conference on Computer Vision and Pattern Recognition.

[24] He K., Zhang X., Ren S., Sun J. Deep residual learning for image recognition. Proceedings of the 2016 IEEE Conference on Computer, Vision, Pattern Recognition.

[25] He K., Zhang X., Ren S., Sun J. (2016). Identity Mapping in deep residual networks. arXiv.

[26] Xie S., Girshick R., Dollár P., Tu Z., He K. (2017). Aggregated Residual Transformations for Deep Neural Networks. arXiv.

[27] Zhang K., Sun M., Han T.X., Yuan X., Guo L., Liu T. (2017). Residual Networks of Residual Networks: Multilevel Residual Networks. IEEE Trans. Circ. Syst. Video Technol..

[28] Zagoruyko S., Komodakis N. (2016). Wide Residual Networks. arXiv.

[29] Huang J., Rathod V., Sun C., Zhu M., Korattikara A., Fathi A., Fischer I., Wojna Z., Song Y., Guadarrama S. Speed/accuracy trade-offs for modern convolutional object detectors. Proceedings of the IEEE Computer Society Conference on Computer Vision and Pattern Recognition.

[30] Ren S., He K., Girshick R., Sun J. (2016). Faster R-CNN: Towards Real-Time Object Detection with Region Proposal Networks. IEEE Trans. Pattern Anal. Mach. Intell..

[31] Liu W., Anguelov D., Erhan D., Szegedy C., Reed S., Fu C., Berg A.C. SSD: Single Shot MultiBox Detector. Proceedings of the European Conference on Computer Vision—ECCV.

[32] Dai J., Li Y., He K., Sun J. (2016). R-FCN: Object Detection via Region-based Fully Convolutional Networks. arXiv.

[33] Irudayaraj J. (2009). Pathogen Sensors. Sensors.

[34] Meroni M., Rosini M., Picchi V., Panigada C., Cogliati S., Nali C., Colombo R. (2008). Asse Assessing Steady-state Fluorescence and PRI from Hyperspectral Proximal Sensing as Early Indicators of Plant Stress: The Case of Ozone Exposure. Sensors.

[35] Wah Liew O., Chong P., Li B., Asundi K. (2008). Signature Optical Cues: Emerging Technologies for Monitoring Plant Health. Sensors.

[36] Mazarei M., Teplova I., Hajimorad M., Stewart C. (2008). Pathogen Phytosensing: Plants to Report Plant Pathogens. Sensors.

[37] Ryant P., Dolezelova E., Fabrik I., Baloum J., Adam V., Babula P., Kizek R. (2008). Electrochemical Determination of Low Molecular Mass Thiols Content in Potatoes (*Solanum tuberosum*) Cultivated in the Presence of Various Sulphur Forms and Infected by Late Blight (*Phytophora infestans*). Sensors.

[38] Dalal N., Trigs B. Histogram of Oriented Gradients for Human Detection. Proceedings of the IEEE Computer Society Conference on Computer Vision and Pattern Recognition.

[39] Lowe D. (2004). Distinctive Image Features from Scale-Invariant Keypoints. Int. J. Comput. Vis..

[40] Cortes C., Vapnik V. (1995). Support Vector Networks. Mach. Learn..

[41] Schapire R. A Brief Introduction to Boosting. Proceedings of the Sixteenth International Joint Conference on Artificial Intelligence.

[42] Pawara P., Okafor E., Surinta O., Schomaker L., Wiering M. Comparing Local Descriptors and Bags of Visual Words to Deep Convolutional Neural Networks for Plant Recognition. Proceedings of the 6th International Conference on Pattern Recognition Applications and Methods (ICPRAM 2017).

[43] Cugu I., Sener E., Erciyes C., Balci B., Akin E., Onal I., Oguz-Akyuz A. (2017). Treelogy: A Novel Tree Classifier Utilizing Deep and Hand-crafted Representations. arXiv.

[44] Amara J., Bouaziz B., Algergawy A. (2017). A Deep Learning-based Approach for Banana Leaf Diseases Classification. Lecture Notes in Informatics (LNI).

[45] Johannes A., Picon A., Alvarez-Gila A., Echazarra J., Rodriguez-Vaamonde S., Diez-Navajas A., Ortiz-Barredo A. (2017). Automatic plant disease diagnosis using mobile capture devices, applied on a wheat use case. Comput. Electron. Agric..

[46] Fujita E., Kawasaki Y., Uga H., Kagiwada S., Iyatomi H. Basic investigation on a robust and practical plant diagnostic system. Proceedings of the 2016 15th IEEE International Conference on Machine Learning and Applications (ICMLA).

[47] Kawasaki Y., Uga H., Kagiwada S., Iyatomi H., Bebis G. (2015). Basic Study of Automated Diagnosis of Viral Plant Diseases Using Convolutional Neural Networks. Advances in Visual Computing, Proceedings of the 11th International Symposium, ISVC 2015, Las Vegas, NV, USA, 14–16 December 2015.

[48] Owomugisha G., Mwebaze E. Machine Learning for Plant Disease Incidence and Severity Measurements from Leaf Images. Proceedings of the 2016 15th IEEE International Conference on Machine Learning and Applications (ICMLA).

[49] Sladojevic S., Arsenovic M., Anderla A., Culibrk D., Stefanovic D. (2016). Deep Neural Networks Based Recognition of Plant Diseases by Leaf Image Classification. Comput. Intell. Neurosci..

[50] Mohanty S.P., Hughes D., Salathe M. (2016). Using Deep Learning for Image-Based Plant Disease Detection. Front. Plant Sci..

[51] Hughes D.P., Salathe M. (2016). An open access repository of images on plant health to enable the development of mobile disease diagnostics. arXiv.

[52] Wang G., Sun Y., Wang J. (2017). Automatic Image-Based Plant Disease Severity Estimation Using Deep Learning. Comput. Intell. Neurosci..

[53] Everingham M., Van Gool L., Williams C., Winn J., Zisserman A. (2010). The Pascal Visual Object Classes (VOC) Challenge. Int. Comput. Vis..

